# Red papules associated with progressive functional decline

**DOI:** 10.1016/j.jdcr.2023.07.020

**Published:** 2023-07-27

**Authors:** David Y. Cao, Jessica Zhou, Carl Dernell, Keri Chaney, Karolyn A. Wanat

**Affiliations:** a School of Medicine, Medical College of Wisconsin, Milwaukee, Wisconsin; b Department of Pathology, Medical College of Wisconsin, Milwaukee, Wisconsin; c Department of Dermatology, Medical College of Wisconsin, Milwaukee, Wisconsin

**Keywords:** hemangiomas, intravascular B-cell lymphoma

## Case

A 77-years-old man with a 4-month history of progressive functional decline presented after a seizure and brief loss of consciousness.

**Fig 1:**
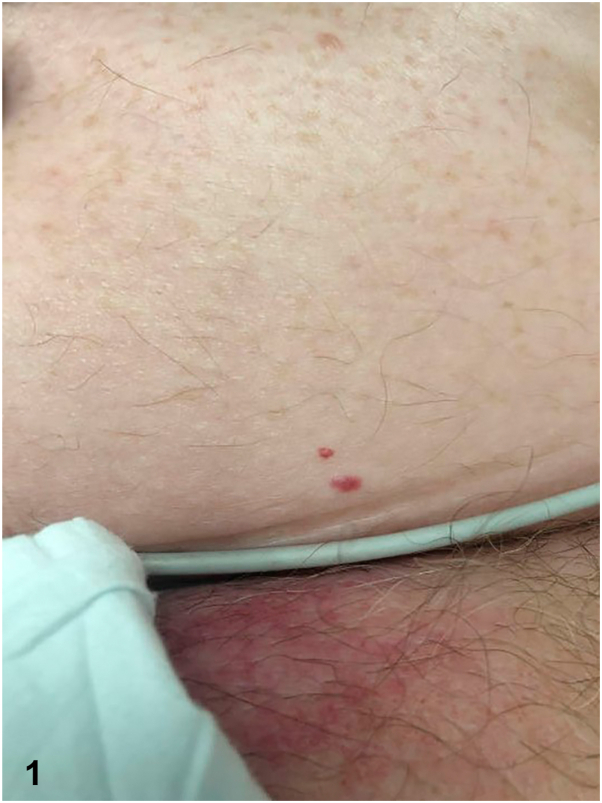

**Fig 2:**
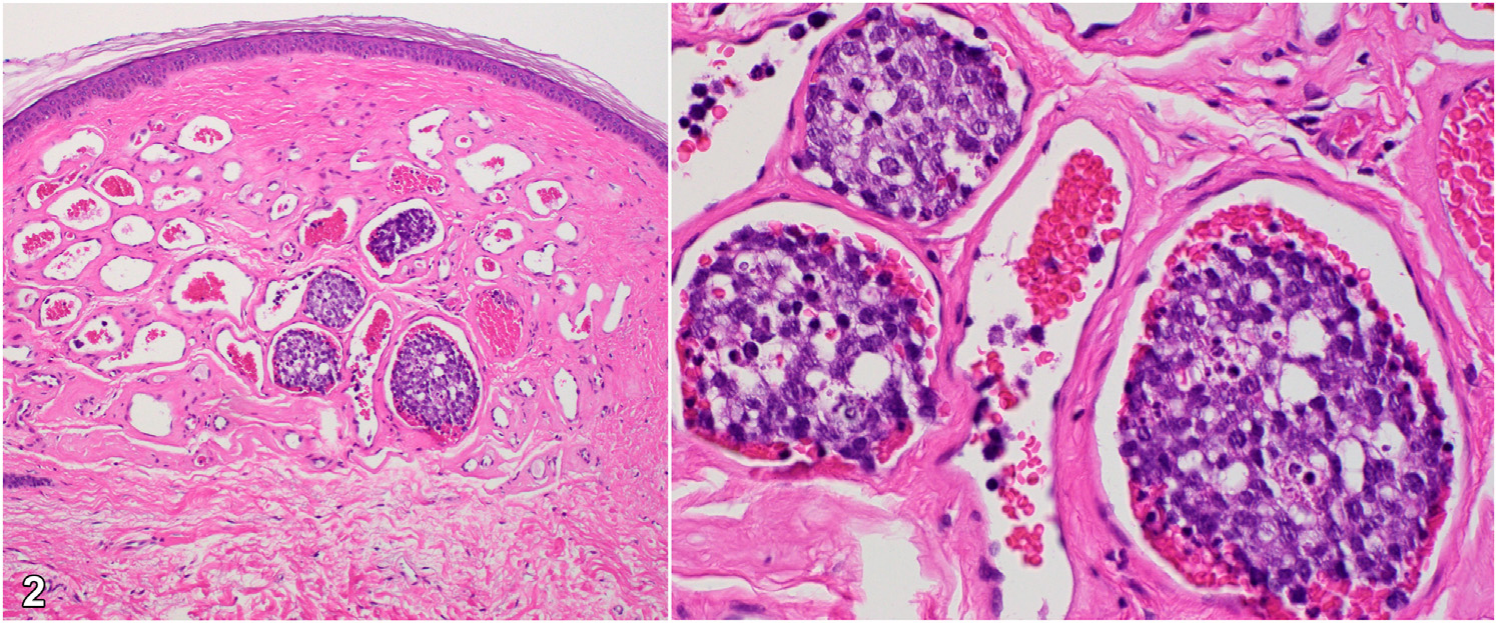

**Fig 3:**
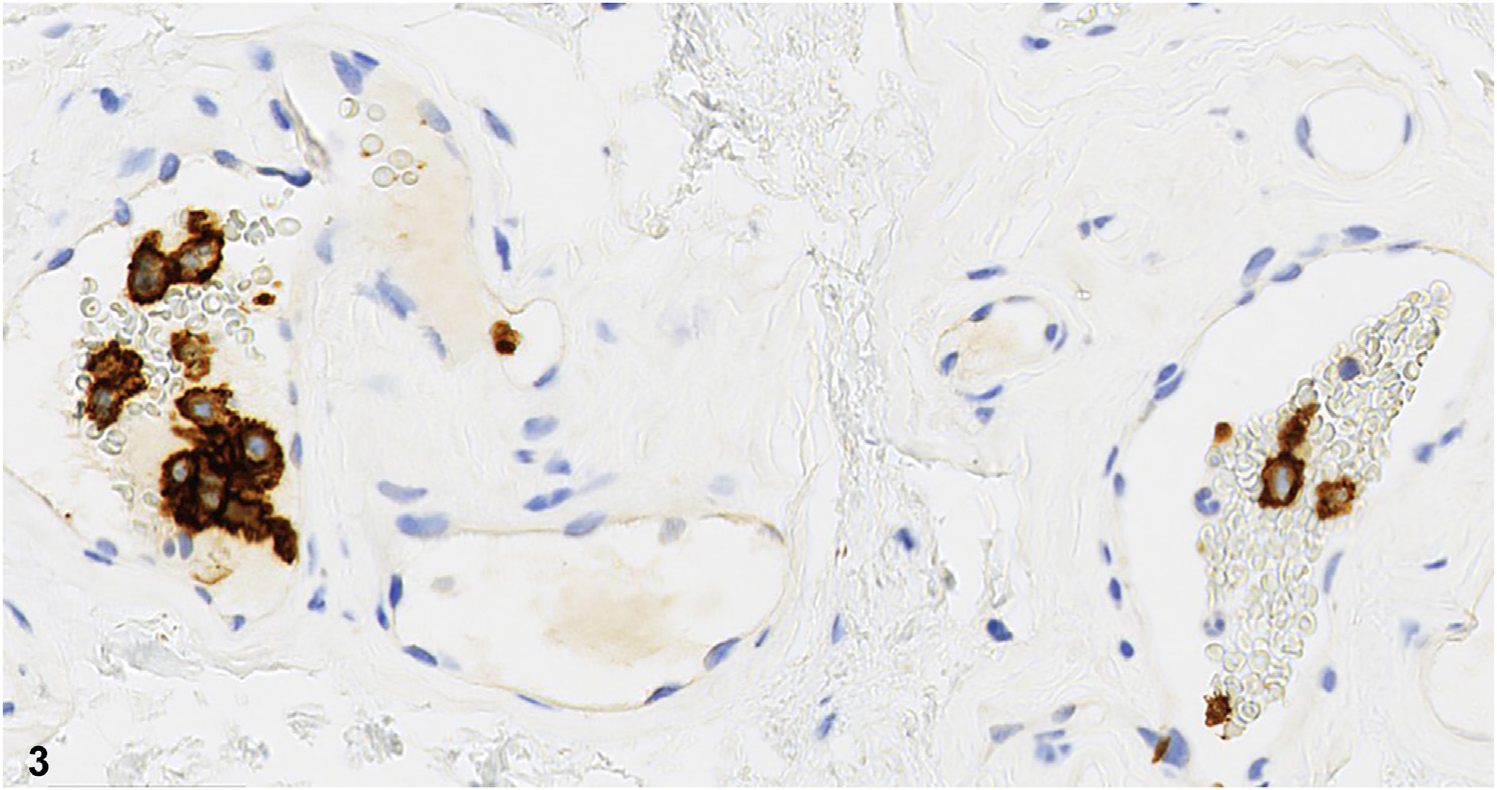

Skin examination revealed red papules on the left abdomen and purpuric patches attributed to idiopathic thrombocytopenic purpura. The red papule was biopsied to help establish a diagnosis (Fig 1). Histopathology demonstrated superficial dermal vessels with intraluminal blood cells and enlarged lymphocytes lined by flattened endothelial cells. H&E (Fig 2) and CD20 immunohistochemical stains (Fig 3) are shown.

Magnetic resonance imaging (MRI) revealed punctate frontal lobe infarcts and hyperintense lower thoracic cord signal with cauda equina involvement. Lumbar puncture showed elevated protein with lymphocyte pleocytosis, and laboratory tests revealed no evidence of lymphoproliferative disorder.

**Question 1: Based on clinical presentation and histopathology, what is the most likely diagnosis?**

A. Eruptive cherry angiomatosis associated with Multicentric Castleman Disease (MCD)
B. Intravascular large B-cell lymphoma (IVLBCL)
C. IgA vasculitis
D. Lymphangioma
E. Kaposi sarcoma

**Answers:**

A. Eruptive cherry angiomatosis associated with Multicentric Castleman Disease (MCD) – Incorrect. Although cherry hemangiomas are present in both MCD and IVLBCL, the hallmark finding of lymphadenopathy in MCD is not present in IVLBCL. The histopathology would demonstrate angiomas without atypical CD20+ cells within the superficial lumens.
B. Intravascular large B-cell lymphoma (IVLBCL) – Correct. IVLBCL is characterized by proliferation of atypical CD20+ lymphoma cells within the lumina of small blood vessels, usually without circulating neoplastic cells or extravascular tumors.^1^ The histopathology demonstrated atypical CD20+ lymphocytes within the superficial vessels of the cherry angioma. Biopsying a cherry angioma can be a helpful way to establish the diagnosis as it can increase the diagnostic yield.^2^
C. IgA vasculitis – Incorrect. While brain infarcts can occur in vasculitides, IgA vasculitis commonly affects children and presents with violaceous papules and pustules on the lower limbs and buttocks. Histopathology would demonstrate fibrinoid necrosis of the superficial vessels with associated leukocytoclasia and red blood extravasation.
D. Lymphangioma – Incorrect. Lymphangiomas are noncancerous fluid-filled cysts in children, usually on the head and neck. Histopathology would demonstrate large lymphatic cisterns that lie deep in the subcutaneous plane and communicate via dilated dermal lymphatic channels lined with endothelial cells.
E. Kaposi sarcoma – Incorrect. Kaposi sarcoma presents with violaceous lesions on the body and oral mucosa but the histopathology of KS demonstrates spindled cells with extravasated red blood cells and hemosiderin deposition; HHV-8 is positive in the spindled cells.^3^

**Question 2: What other cutaneous lesions have been reported and could present with this condition?**

A. Subcutaneous fluid-filled cyst development on the cheek or neck
B. Bilateral leg edema
C. Violaceous papules and plaques on the oral mucosa
D. Purpuric papules and pustules with associated ulcerations
E. Nodules and plaques on the trunk or limbs

**Answers:**

A. Subcutaneous fluid-filled cyst development on the cheek or neck – Incorrect. Fluid-filled cysts on the face and neck are characteristic of lymphangioma.
B. Bilateral leg edema – Incorrect. IVLBCL is associated with diffuse, anasarca-like edema of the face, body, and abdomen.^1^^,^^4^
C. Violaceous papules and plaques on the oral mucosa – Incorrect. Violaceous spots of the oral mucosa are most closely associated with Kaposi sarcoma.
D. Purpuric papules and pustules with associated ulcerations – Incorrect. Purpuric papules and pustules with ulcerations are most closely associated with vasculitis.
E. Nodules and plaques on the trunk or limbs – Correct. Forty percent of patients with IVLBCL present with cutaneous features, and of those patients, 49% present with nodules and plaques of the trunk and limbs.^1^ Other cutaneous findings can include cellulitis, ulcerated nodules, telangiectasia, and erythematous eruptions.^4^

**Question 3: What is the overall prognosis and treatment of this condition?**

A. Poor prognosis that should be promptly treated with ruxolitinib
B. Poor prognosis that should be promptly treated with R-CHOP (rituximab, cyclophosphamide, hydroxydoxorubicin, vincristine, and prednisone) chemotherapy combination
C. Good prognosis that can be further improved by RICE (rituximab, ifosfamide, carboplatin, etoposide) chemotherapy combination
D. Good prognosis that can be further improved by HAART (highly active antiretroviral therapy) agents and chemotherapy combination – such as lamivudine and ritonavir with liposomal doxorubicin or paclitaxel
E. Good prognosis that self-resolves, including the skin lesions

**Answers:**

A. Poor prognosis that should be promptly treated with ruxolitinib – Incorrect. Although IVLBCL has a poor prognosis, ruxolitinib is not used as a treatment for IVLBCL. Ruxolitinib is an appropriate treatment for myeloproliferative disorders, such as myelofibrosis and polycythemia vera.
B. Poor prognosis that should be promptly treated with R-CHOP (rituximab, cyclophosphamide, hydroxydoxorubicin, vincristine, and prednisone) chemotherapy combination – Correct. More than 80% of patients with IVLBCL treated with rituximab-containing combination chemotherapy such as R-CHOP (rituximab, cyclophosphamide, hydroxydoxorubicin, vincristine, and prednisone) achieve complete remission.^1^^,^^4^^,^^5^
C. Good prognosis that can be further improved by RICE (rituximab, ifosfamide, carboplatin, etoposide) chemotherapy combination – Incorrect. IVLBCL has a poor prognosis, and although RICE chemotherapy includes rituximab (an anti-CD20 monoclonal antibody), the ICE combination (ifosfamide, carboplatin, and etoposide) is not the most effective treatment for IVLBCL.
D. Good prognosis that can be further improved by HAART (highly active antiretroviral therapy) agents and chemotherapy combination – such as lamivudine and ritonavir with liposomal doxorubicin or paclitaxel – Incorrect. Although the HAART/chemotherapy combination contains chemotherapy drugs that may treat IVLBCL, the most effective treatment for IVLBCL is R-CHOP. HAART agents with chemotherapy are an appropriate treatment for Kaposi sarcoma.^3^
E. Good prognosis that self-resolves, including the skin lesions – Incorrect. IVLBCL has a poor prognosis, and skin lesions may remain or worsen (do not self-resolve).

## Conflicts of interest

None disclosed.
